# The effect of the SIMS Programme versus existing preschool oral healthcare programme on oral hygiene level of preschool children: study protocol for a cluster randomised controlled trial

**DOI:** 10.1186/s13063-021-05111-0

**Published:** 2021-02-22

**Authors:** Zamros Yuzadi Mohd Yusof, Nurul Hayati Anwar, Nor Azlida Mohd Nor, Mariani Md Nor, Siti Ezaleila Mustafa

**Affiliations:** 1 Department of Community Oral Health & Clinical Prevention, Faculty of Dentistry, University of Malaya, Kuala Lumpur, Malaysia; 2 Community Oral Health Research Group, Faculty of Dentistry, University of Malaya, Kuala Lumpur, Malaysia; 3 Oral Health Division, Terengganu Health Department, Terengganu, Malaysia; 4 Department of Educational Psychology & Counselling, Faculty of Education, University of Malaya, Kuala Lumpur, Malaysia; 5 Department of Media and Communication Studies, Faculty of Arts and Social Sciences, University of Malaya, Kuala Lumpur, Malaysia

**Keywords:** Dental plaque, Oral hygiene, Preschool children, Malaysia, Oral health behaviour, SIMSP, Oral health literacy

## Abstract

**Background:**

Despite the implementation of the preschool oral healthcare programme (POHP) for 5–6-year-old children over the past 3 decades in Malaysia, dental plaque and caries levels in this age group remain high. Among the child-level attributable factors are unhealthy self-care behaviours (poor oral hygiene and high sugary diet). In order to improve the children’s oral health, an improved programme called the ‘Senyuman Indah Milik Semua’ Programme (SIMSP) or ‘Beautiful Smile for All’ programme is introduced. In this programme, a triad of dental hygienist-teacher-parent works together to improve children’s oral hygiene levels compared with the existing POHP that involves dental hygienists only. The aim of this study is to compare the effect of the SIMSP versus the existing POHP on oral hygiene levels of 5–6-year-old children in the Kampar district, Perak state, Malaysia.

**Methods:**

This study is a pragmatic, cluster-randomised, parallel-group, matched pair, controlled trial with blinded outcome assessment. Randomisation is performed using a computer-generated table with a 1:1 allocation comparing the SIMSP and the POHP involving 28 preschools in the Kampar district, Perak, Malaysia. The intervention consists of preschool visits by a group of dental therapists, in-class oral health lessons and daily toothbrushing conducted by class teacher, child home toothbrushing supervised by parents, and infographic oral health messages to parents. The control consists of the existing POHP that involves preschool visits by a group of dental therapists only. The trial lasts for 6 months. Primary outcome variable is the mean plaque score change after 6 months. To determine the feasibility of the SIMSP, a process evaluation will be conducted using the perspectives of dental therapists, teachers, and parents on the appropriateness, effectiveness, facilitators, and barriers to the SIMSP implementation as well as an audit trail to assess the trial intervention.

**Discussion:**

Cluster randomisation may lead to a random effect and cluster selection bias. These factors will be accounted for when analysing the data and interpreting the outcomes. The effectiveness of the SIMSP will be evaluated by comparing the results with those of the POHP.

**Trial registration:**

ClinicalTrials.gov NCT04339647. Registered on 5 April 2020 – Retrospectively registered.

## Administrative information

The order of the items has been modified to group similar items (see http://www.equator-network.org/reporting-guidelines/spirit-2013-statement-defining-standard-protocol-items-for-clinical-trials/).

**Table:** 

Title {1}	**The effect of the SIMS Programme versus existing preschool oral healthcare programme on oral hygiene level of preschool children: study protocol for a cluster randomised controlled trial**
Trial registration {2a and 2b}.	ClinicalTrials.gov ID: NCT04339647
Protocol version {3}	5 April 2020, 1st version.
Funding {4}	Impact-oriented Interdisciplinary Research Grant (IIRG-035A-2019), University of Malaya
Author details {5a}	Zamros Yuzadi Mohd Yusof ^1,2^ Nurul Hayati Anwar ^1,2,3^, Nor Azlida Mohd Nor ^1,2^, Mariani Md Nor^4^, Siti Ezaleila Mustafa^5^ ^1^ Department of Community Oral Health and Clinical Prevention, Faculty of Dentistry, University of Malaya, Kuala Lumpur, Malaysia. ^2^ Community Oral Health Research Group, Faculty of Dentistry, University of Malaya, Kuala Lumpur, Malaysia. ^3^ Oral health division, Perak Health Department, Malaysia. ^4^ Department of Educational Psychology & Counselling, Faculty of Education, University of Malaya, Kuala Lumpur, Malaysia. ^5^ Department of Media and Communication Studies, Faculty of Arts and Social Sciences, University of Malaya, Kuala Lumpur, Malaysia. ZYMY is the chief investigator and conceived the study. ZYMY and NAMN led the SIMSP proposal and protocol development. NHA, MMN and SE contributed to the study design and development of the proposal. ZYMY is the lead trial methodologist. NHA led the development of the SIMSP packages. ZYMY, MMN, NAMN and SE contributed towards the development of the intervention packages. NHA provided advice on recruitment strategies, participant information literature, and school and parental engagement strategies. ZYMY drafted the manuscript. All authors read and approved the final manuscript.
Name and contact information for the trial sponsor {5b}	**Trial Sponsor:** University of Malaya. **Sponsor’s Reference:** IIRG-035A-2019. **Contact name:** Ainin Sulaiman. **Address:** Dean, Social Advancement and Happiness Research Cluster, Level 7, Research Management & Innovation Complex (RMIC), University of Malaya, 50603 Kuala Lumpur, MALAYSIA.**Telephone:** Tel: +603-7967 7802 / 7804 / 7809. Fax: +603-7967 7813. Email: researchcluster@um.edu.my
Role of sponsor {5c}	This funding source has no role in the design of the trial and will not have any role during its execution, analyses, interpretation of data, or decision to submit results.

## Introduction

### Background and rationale {6a}

Oral disease is one of the costliest diet and lifestyle-related diseases [1]. Many oral health problems can be prevented and their early onset reversible. However, in some countries, there are a large number of children and parents who have limited knowledge on the causes and prevention of oral disease [2].

In Malaysia, preschool children are one of the target groups in the delivery of dental services by the Ministry of Health, Malaysia [3]. The preschool oral healthcare programme (POHP) was introduced in 1984 to provide preventive and curative treatment as well as oral health education (OHE) to preschool children aged 5–6 years [3]. The POHP is delivered by dental therapists (DTs) who visit preschools twice a year. In the first visit, DTs will conduct an oral examination followed by OHE using either role play, puppet show, video presentation, or tell-show-do technique. On the same visit, a toothbrushing exercise and a fluoride varnish application are carried out. The second visit takes place after 6 months where DTs will perform atraumatic restorative treatment [4] using glass ionomer restorations followed by a second application of fluoride varnish. Apart from preschool children, DTs also provide oral healthcare to primary and secondary school children, children with special needs, elderly people, and provide OHE to selected government agencies and health centres [5].

Although the POHP covers most preschools in Malaysia for the past 3 decades except private preschools, the prevalence of caries and children with dental plaque in this age group remain high. The national oral health data in 2005 showed that 76.2% (mean dft = 5.5) of 5-year-old children had caries in the primary teeth [6] and the prevalence reduced to 71.3% (mean dft = 4.8) after 10 years [7]. Both prevalences are well over the national target of 50% caries in this age group by 2020 [8]. In comparison, caries prevalence in this age group was slightly lower than the median caries prevalence of 79% among 5–6-year-old children in Southeast Asia in 2017 (mean dft = 5.1) [9]. The limitations of the current POHP in controlling caries and dental plaque in young children had been highlighted in the 2015 national oral health survey report [7]. Among the attributable factors highlighted were limited resources, lack of time, wider job scopes of DTs, and lack of parental and teachers’ involvement in children oral health. All these led to poor self-care behaviours of children.

In order to improve the POHP and to prevent preschool children from developing future caries in permanent dentition, positive changes in children’s self-care behaviours are crucial [10]. Positive changes in oral health behaviours would lead to long term reductions in dental plaque and dental caries. As children live with parents and attend school, positive changes in oral health behaviours in this age group would invariably require the combined efforts of DTs, parents and teachers in children’s oral health [11, 12].

Based on the 2015 survey findings, the recommendations put forward to improve the existing POHP are to strengthen the DT’s role in preschool oral health, empower parents, and include teachers in OHE. The use of social media in OHE is also recommended [13]. As the result, the Senyuman Indah Milik Semua Programme (SIMSP) or Beautiful Smile for All Programme was introduced in 2019 taking into account scientific evidence, best practice, and stakeholders’ feedback. In addition to the DT’s role in current POHP, the SIMSP will also include active participation of teachers and parents. The details of the SIMSP are described in the “Methods: participants, interventions, and outcomes” section.

### Scientific hypothesis

The scientific hypothesis of the study is that the SIMSP is more effective than the POHP to improve preschool children’s oral hygiene after 6 months. If it is proven effective, the SIMSP may be absorbed into the current oral healthcare service to replace the POHP.

### Objectives {7}

The primary objective of the study is to assess the effect of the SIMSP versus existing POHP on mean plaque score change among 5–6-year-old children over 6 months.

The secondary objectives are:

1) To assess the impact of the SIMSP versus existing POHP over 6 months in terms of:

- Changes in children’s oral health behaviours
- Mean change in parental oral health literacy (OHL) score

2) To undertake a process evaluation using the perspectives of DTs, teachers, and parents on the appropriateness, effectiveness, facilitators, and barriers to the implementation of the SIMSP intervention.

#### Hypothesis (alternative)

1. The SIMSP is effective to reduce plaque scores among 5–6-year-old children compared to existing POHP.
2. The SIMSP is effective to promote positive changes in oral health behaviours among 5–6-year-old children compared to existing POHP.
3. The SIMSP is effective to improve parental OHL scores compared to existing POHP.

### Trial design {8}

The study design is a pragmatic, cluster-randomised, parallel-group, matched pair, controlled trial with blinded outcome assessment.

## Methods: participants, interventions, and outcomes

### Study setting {9}

The trial will be centred at government-funded preschools in Kampar district, Perak state, Malaysia. There are 53 preschools altogether, and the preschools are paired according to geographical location and preschool characteristics into 24 pairs (5 preschools are not paired as their locations are far away from each other).

### Eligibility criteria {10}

The inclusion criteria for preschool are government-funded preschool that receives the POHP. The inclusion criteria for children are healthy children aged 5–6 years, understand Malay language, live with parents/caregivers, and have a written informed consent. For parents/caregivers, they must be fluent in Malay language and live with the child.

Children with chronic medical conditions, dental/oral developmental conditions, physical disability or long term medication are excluded.

### Who will take informed consent? {26a}

DTs will inform parents about the study in a letter to be sent through school teacher. The letter contains information sheets about the study and a consent form. A contact telephone number is included in the consent form for parents to call the DTs if they have any questions about the study. A written informed consent from parents will be obtained before their child can be included in the study.

### Additional consent provisions for collection and use of participant data and biological specimens {26b}

A verbal consent from children will be obtained before their teeth are examined.

### Interventions

#### Explanation for the choice of comparators {6b}

Preschool children in the control group will receive the existing POHP provided by the Oral Health Programme, Ministry of Health. In the POHP, a team of DTs will visit the preschool twice/year. The activities will consist of an oral examination, OHE including toothbrushing exercise, fluoride varnish application twice/year, and simple treatment using glass ionomer restorations (if required).

#### Intervention description {11a}

The SIMSP was developed based on feedback from experts in dental public health discipline, senior dental officers in the Ministry of Health, preschool curriculum experts in the Ministry of Education, preschool teachers, a child psychologist, and parents. The concept of the SIMSP is that improvement in preschool children’s oral hygiene would require the combined efforts of DTs, preschool teachers, and parents working together on children’s oral health. The SIMSP’s target groups are preschool children and their parents. It consists of the following package:

1) Preschool children:

- Oral examination, OHE, fluoride varnish application twice/year, and simple treatment by DTs (usual care/POHP);
- In-class oral health lessons by teacher based on the teacher’s OHE booklet over a period of 6 months;
- In-school daily toothbrushing with fluoride toothpaste (1450 ppm fluoride) supervised by class teacher for 6 months;
- Supervised home toothbrushing at night by parents/guardians.

2) Parents/guardians:

- Attend a parent-DT meeting at school to discuss on child’s caries risk assessment (CRA);
- Received OHE and diet advice from DT based on child’s CRA levels (low/medium/high);
- Received free toothbrush and fluoride toothpaste (1450 ppm fluoride) for child home toothbrushing;
- Received 10, 2-weekly oral health infographics from DT sent via electronic messaging application (WhatsApp) for a duration of 5 months (printed versions for parents without a smartphone).

#### Teacher’s OHE booklet

The OHE booklet was developed prior to the study for use by teachers as a teaching tool for in-class oral health lessons. The content was developed by experts in dental public health discipline based on thorough review of dental literature. It consists of 6 oral health domains with 11 topics. The first domain covers basic knowledge about teeth with 4 topics: (i) your teeth, (ii) healthy teeth and their use, (iii) milk and permanent teeth, and (iv) why my teeth are loose? The second domain covers basic knowledge on toothbrushing with 1 topic: Toothbrushing is fun. The third and fourth domains promote children’s awareness on oral disease with 2 topics: (i) dental caries and (ii) gum disease, respectively. The fifth domain provides awareness on sugary diet with 1 topic: Sugars are bad for teeth. The sixth domain teaches about self-motivation with 3 topics: (i) you can do it, (ii) going to the dentist, and (iii) beautiful smile for all.

The OHE booklet was content validated by a paediatric dentist, a periodontist, and a general dentist. The delivery method and the level of language used were assessed and verified by a child psychologist. Subsequently, preschool teachers’ feedback was sought on the overall appearance of the booklet and the suitability of the worksheets for preschool children use. The booklet was tested on a group of preschool children who were not involved in the main study to assess its utility as a teaching tool before it was finalised. It was subsequently endorsed by the Committee on Preschool Curriculum from the Ministry of Education who were experts in preschool pedagogy and assessment before being used in the study. Teachers in the SIMSP will deliver oral health lessons based on the OHE booklet every 2 weeks for 6 months. Each lesson will take approximately 20 to 30 min. At the end of each lesson, a revision in the form of a colouring worksheet will be distributed to the children as part of the learning activities.

#### Parent's oral health infographics

The parents/caregivers’ oral health infographics were developed prior to the study. The infographics consist of 5 domains with 10 topics related to oral health. The content was developed by experts in dental public health discipline and validated by a paediatric dentist, a periodontist, and a social media expert. The first domain has 1 topic on knowledge of teeth structure and eruption dates. The second domain relates to oral health habits with 2 topics: (i) good oral health habits and parental roles in children’s oral health, and (ii) poor oral health habits. The third domain covers knowledge on oral disease with 2 topics: (i) dental caries, and (ii) periodontal disease. The fourth domain relates to toothbrushing with fluoride toothpaste with 2 topics: (i) toothbrushing, and (ii) use of fluoride toothpaste. The fifth domain contains information on dental treatment and prevention with 3 topics: (i) fluoride varnish, (ii) fissure sealant, and (iii) treatment of oral disease. The infographics were tested with a group of preschool parents. Their opinions for improvement were sought before the infographics were finalised. In the SIMSP intervention, the infographics will be sent to parents/guardians every 2 weeks over a period of 5 months.

#### Caries risk assessment (CRA) form

The CRA form used in this study was adapted from the CRA template developed by experts during the Malaysia Early Caries Expert Workshop 2014. It consists of 4 parts taking into account the clinical, environmental, behavioural, and factors associated with parents/caregivers [14]. Part 1 contains information on child’s caries experience assessed by DTs using the International Caries Detection and Assessment System (ICDAS) [15]; part 2 contains information on caries risk factors of the child: presence of visible plaque, use of fluoride toothpaste, presence of crowding/deep fissures, consumption of sugary snacks, bottle feeding at night, and mother/siblings with caries (visible plaque and crowding/deep fissures were assessed together in Part 1); part 3 contains information on the caries risk indicator of the child, i.e. low, medium, or high based on information in parts 1 and 2; part 4 contains information on caries management of the child including individual treatment plan, and tailored OHE and dietary advice given to parents during parent-DT meeting.

### Control

Preschool children in the control group received the usual care/POHP delivered by DTs in 2 visits/year. It consists of an oral examination, OHE (including toothbrushing exercise), fluoride varnish application twice/year, and simple treatment.

### Implementation of the intervention

**Fig. 1:**
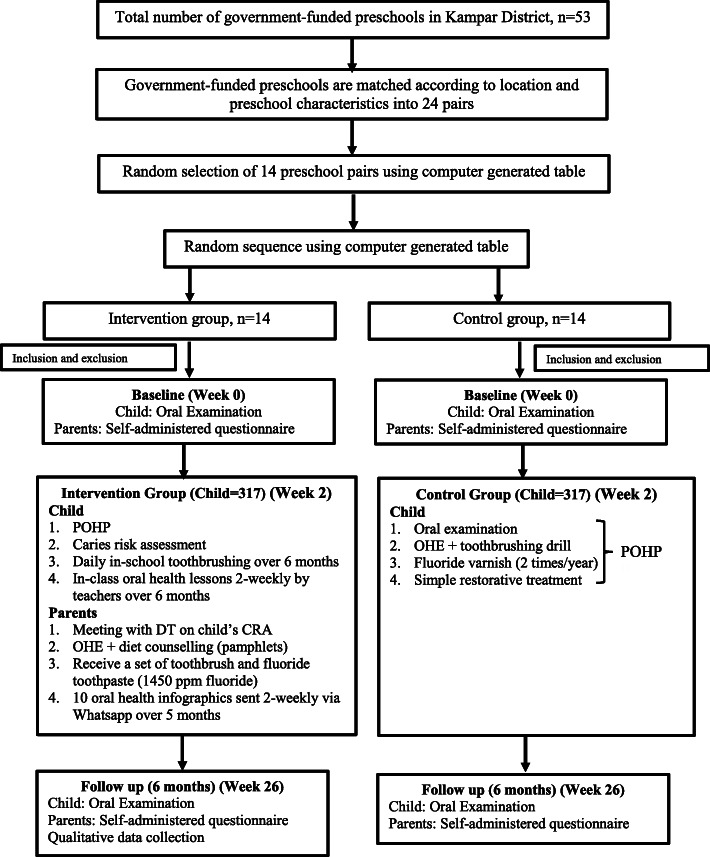
Timeline of the study

The SIMSP intervention is delivered for a period of 6 months (Fig. 1). It consists of 3 phases:

#### Phase 1: DT first visit to preschools in both groups

A written informed consent from parents for their children’s involvement in trial will be sought when the preschools open in the new academic year. The first visit of DTs to preschools occurs in the beginning of the year to conduct an oral examination of the children for caries and dental plaque. In the SIMSP group, the presence of crowding/deep fissures on the teeth will be charted and this information will be included in the CRA form. A self-administered questionnaire for parents will be sent through class teacher.

#### Phase 2: DT second visit to preschools and initiation of intervention

This visit takes place 2 weeks after the first visit. In the SIMSP group, DTs will deliver a standardised OHE to children followed by a fluoride varnish (20,000 ppm fluoride) application on their teeth. At this visit, DTs will meet parents to discuss their child’s oral health status and fill in the child’s CRA form. Parents are given OHE and dietary advice based on their child’s caries risk levels (low/medium/high). A set of free toothbrush and fluoride toothpaste (1450 ppm fluoride) is given to parents for child home toothbrushing along with instructions. Parental agreement to receive 10 oral health infographics via WhatsApp every 2 weeks for the next 5 months will be sought. With regards to teachers, they will receive the Teacher OHE booklet to be used as a teaching aid for teaching oral health lessons in class. Teachers will also be given enough supplies of toothbrush and fluoride toothpaste for children’s daily toothbrushing over 6 months. Teachers are given instructions on toothbrushing and a teeth model to help them with the activity. In the control group, DTs will deliver the POHP that consists of a standardised OHE (including toothbrushing exercise) followed by a fluoride varnish (20,000 ppm fluoride) application on children’s teeth.

#### Phase 3: DT third visit to preschools

This visit will take place 24 weeks after the second visit. In this visit, DTs will perform simple dental treatment on the children (if required) followed by the second application of fluoride varnish (20,000 ppm fluoride). Children in the SIMSP and POHP groups will receive the same treatment on the third visit.

### Criteria for discontinuing or modifying allocated interventions {11b}

This section is not relevant as children in both groups will not receive any drug prescriptions except fluoridated toothpaste and fluoride varnish application.

### Strategies to improve adherence to interventions {11c}

A face-to-face instructions to parents will take place during parent-DT meeting at the beginning of the year. This session will include:

- Instructions on child home toothbrushing.
- The importance of reducing child’s daily sugar intake and practising healthy bottle-feeding pattern.

A reminder will also be sent via WhatsApp messaging platform every month.

For teachers, face-to-face adherence reminder sessions will take place at the beginning of the intervention at school and monthly for 6 months. This session will include:

- Revision on the delivery of the OHE lessons in class
- Revision on children’s daily toothbrushing supervision at school.

Procedures for monitoring adherence will involve several other methods; in-class lessons by filling in the lesson dates in the booklet by teacher, in-school toothbrushing by completing a toothbrushing diary by teacher, parental meeting with DTs by completing the attendance list by DTs, and infographics sent to parents by completing a standardised report form by DTs. At the same time, feedback from teachers, DTs, and parents will be gathered by means of focus group discussion (FGD) (with parents and DTs) and in-depth interview (with parents) to assess the process implementation of the SIMSP intervention.

### Relevant concomitant care permitted or prohibited during the trial {11d}

The children are allowed to visit the dentist and receive treatment for any dental problems they face during the study period. Cases of non-adherence or deviation from the study protocol will be reported. Their data may not be included in the analysis.

### Provisions for post-trial care {30}

This aspect is not relevant as there is no ancillary or post-trial care provided to the children.

### Outcomes {12}

The primary outcome variable is the mean plaque score change in children after 6 months assessed using the Oral Cleanliness Index [16]. The secondary outcomes are: (1) changes in the children’s oral health behaviours after 6 months, (2) mean change in parental OHL score after 6 months, and (3) qualitative data on the process evaluation of the SIMSP using the perspectives of DTs, teachers, and parents in terms of the appropriateness, effectiveness, facilitators, and barriers to the SIMSP implementation.

### Participant timeline {13}

Data collection takes place at baseline and after 6 months in the intervention and control group. It involves an oral examination of the children and a self-administered questionnaire for parents.

- Oral examination

All children will undergo an oral examination for assessment of dental caries and plaque score at baseline and after 6 months by 3 dental officers after undergoing training and calibration of the examination criteria at the Faculty of Dentistry, University of Malaya. Both examiners will be blinded to the intervention group.

#### Dental caries status

Dental caries will be assessed using the International Caries Detection and Assessment System (ICDAS) [15] which allows for the detection and assessment of caries experience in children. Although caries is not one of the outcome measures, its assessment is taken as a proxy to show both groups are equal in terms of oral disease level at baseline.

#### Plaque score

Plaque score is assessed using the Oral Cleanliness Index which is a modification from the Silness and Loe Plaque Index for assessment of plaque [16]. Assessment for the presence of visible plaque will involve examining the labial surfaces of upper anterior teeth segments from upper right to upper left primary canines. Each surface is recorded using the following codes: 0—teeth appear clean, 1—presence of plaque around the labial cervical margins and covering less than ½ of labial tooth surface, 2—plaque covering more than ½ of labial tooth surfaces, and 9—assessment cannot be made.

b) Parents/guardians questionnaire

The questionnaire consists of 3 sections. Section A consists of items on the socio-demographic characteristics of parents (age, relationship with child, education level, total household income, and family status). Section B consists of items on child’s oral health-related behaviours (tooth brushing, use of fluoride toothpaste, bottle feeding, sugary food and drinks consumption, history of dental visit, and fluoride varnish application). Section C consists of the Malay version of Dental Health Literacy Assessment Instrument (Malay-DHLAI) [17] which consists of 3 parts: Oral Health Knowledge (OHK) domain (12 items), Comprehension domain (5 items), and Skills and Motivation (SM) domain (39 items). The OHK domain contains items on basic dental knowledge, knowledge on oral health promotion, oral health protection, disease prevention and dental visit. Each item is assessed by one correct answer from 4 answer options with total score ranged from 0 to 12. The Comprehension domain contains items on parents/guardians’ ability to understand healthcare instructions using their comprehension skill. Each item is scored by true/false answer options with total score ranged from 0 to 5. The SM domain assesses parents/guardians’ perceptions on their skills and motivation for child’s oral healthcare. Each item is assessed using a 5-point Likert scale (strongly disagree to strongly agree) with total score ranged from 0 to 39.

#### Qualitative data collection

FGD with DTs and teachers will be conducted separately to assess the implementation process of the SIMSP. The interview grid will be developed by identifying essential domains from the literature for evaluating the implementation process of the SIMPS’ protocol. The identified domains are ‘appropriateness’, ‘effectiveness’, ‘facilitators’, and ‘barriers’ to the implementation of the SIMSP. The domains will be included in the interview grid using semi structured open-ended questions to obtain participants’ feedback during FGD. The interview grid with the questions will be tested prior to its use in the study. An in-depth interview with parents will be conducted using the same interview grid. This method is suitable for parents as they have different work commitments. The FGD and in-depth interviews will be conducted by experts who are trained in qualitative methodology. Data collection is expected to last for 1.5 months. For FGD, all DTs and teachers will be included. For in-depth interview, data collection will continue until data saturation has been reached.

##### Sample size {14}

Sample size calculation is based on the effect of the SIMSP on plaque score reduction of the children compared to the POHP after 6 months with a small effect size dz. = 0.30, α = 0.05, power = 0.8. Using G*Power version 3.1.9.2 software, the total number of sample is *n* = 352 (176 per group). This number is increased by 20% to account for non-respondents [18], and multiplied by a design effect of 1.5 to produce the final sample size, *n* = [352 + (352*0.2)] × 1.5 = 634 (317 child per group).

##### Recruitment {15}

The study sample consists of children who attend preschools on weekdays. As a result, enrolment of eligible children will not pose a problem provided parental consent is obtained.

### Assignment of interventions: allocation

#### Sequence generation {16a}

The 53 preschools in the Kampar district are paired according to geographical location and preschool characteristics into 24 pairs (5 preschools are not paired as their locations are far away from each other). In this study, a two-stage randomisation of preschools will be applied. In the first stage, 14 pairs of preschools are randomly selected using computer generated table. In the second stage, using similar method, preschools in each pair will be randomly allocated into intervention and control group with a 1:1 allocation.

#### Concealment mechanism {16b}

The allocation concealment is achieved by having a senior dental officer in the Kampar district who is not involved in the study to keep the allocation sequence in a brown envelope. The information will be kept safe until the preschool pairs have been recruited into the study.

#### Implementation {16c}

The allocation sequence of the preschools will be generated by the statistician employed at the Faculty of Dentistry, University of Malaya using computer-generated table. The information will be kept confidential until the interventions are assigned to the preschools. The children in the preschools will be enrolled by the DT team who will be in charge of implementing the intervention.

### Assignment of interventions: blinding

#### Who will be blinded {17a}

Both examiners will be blinded to the intervention group and do not know which preschools are allocated to intervention and control group.

#### Procedure for unblinding if needed {17b}

Not relevant.

## Data collection and management

### Plans for assessment and collection of outcomes {18a}

#### Plans to promote participant retention and complete follow-up {18b}

Parents will be informed in a letter given through the school teacher whose content includes the importance of completing the questionnaire at the follow-up in 6 months. For children, the follow-up examination will be done at the preschool. Therefore, information on the follow-up examination dates will be included in the letter to parents to ensure their children will attend school on those dates.

#### Data management {19}

Quantitative data will be entered and analysed using the Statistical Package for Social Sciences (SPSS) version 21.0 software (IBM Corp, Armonk, NY, SA). This may be done at the participating sites where the data originated or at the Faculty of Dentistry, University of Malaya. Participant data will be stored in numerical order in files and will be placed in locked research facility at the Faculty of Dentistry, University of Malaya, 50603, Kuala Lumpur, Malaysia for a period of 3 years after completion of the study.

#### Confidentiality {27}

Data will be kept at the strictest confidentiality in a locked research facility at the Faculty of Dentistry, University of Malaya, 50603, Kuala Lumpur, Malaysia. Data may be shared upon request and is subjected to the data protection regulations.

#### Plans for collection, laboratory evaluation and storage of biological specimens for genetic or molecular analysis in this trial/future use {33}

Not relevant.

### Statistical methods

#### Statistical methods for primary and secondary outcomes {20a}

Quantitative data will be entered and analysed using the Statistical Package for Social Sciences (SPSS) version 21.0 software (IBM Corp, Armonk, NY, USA). Both continuous and categorical variables are initially analysed using exploratory data analysis to assess for missing data, data entry errors, as well as using other techniques for testing data assumptions. The preschool is the unit of randomisation and the child is the unit of analysis. Cluster sampling analysis to address cluster effects will be carried out. Intraclass correlation for each outcome variable will be calculated among children in the cluster and among clusters in the district. The effect of the SIMSP at individual and preschool level are analysed. Between-group differences in the primary and secondary outcome variables at baseline and after 6 months will be assessed and compared. Outcomes of the FGD and the in-depth interviews are transcribed verbatim and thematically analysed using coding techniques commonly used in qualitative studies [19].

#### Interim analyses {21b}

No interim-analysis will be performed in the trial.

#### Methods for additional analyses (e.g. subgroup analyses) {20b}

No additional analyses will be conducted in the trial.

#### Methods in analysis to handle protocol non-adherence and any statistical methods to handle missing data {20c}

Intention-to-treat analysis [15] will be carried out for the primary and secondary outcome measures in the study [20].

#### Plans to give access to the full protocol, participant level-data and statistical code {31c}

Data from the trial may be shared upon request. All data are subjected to the data protection regulations.

### Oversight and monitoring

#### Composition of the coordinating centre and trial steering committee {5d}

##### Principal Investigator (ZYMY)

Design of the trial

Preparation of protocol and revisions

Chairing the steering committee meetings

Publication of study reports

##### Steering committee (NHA, NAMN, MMN, SEM)

Agreement of final protocol

Recruitment of preschools and children

Reviewing progress of study to facilitate the smooth running of the study.

Liaise with the dental therapists, school teachers, and parents

##### Composition of the data monitoring committee, its role and reporting structure {21a}

There is no formal data monitoring committee appointed in the trial. The research team will monitor the trial implementation with the help of the DTs. Data will be collected by examiners who are blinded to the intervention. Data entry will be done by a person who is blinded to the intervention. All the personnel involved in the trial are independent from the sponsor and have no competing interests.

##### Adverse event reporting and harms {22}

The trial will not involve administration of drugs to the children. However, any accidents occurred to the children will be recorded and assessed if the causes are related to the trial.

##### Frequency and plans for auditing trial conduct {23}

The researchers will observe the implementation fidelity of the SIMSP to ensure that it is delivered as planned throughout the 6-month period. Data on implementation fidelity are collected by the researchers through various methods; in-class lessons by filling in the lesson dates in the booklet by teacher, in-school toothbrushing by completing a toothbrushing diary by teacher, parental meeting with DT by completing the attendance list by DTs, and infographics sent to parents by completing a standardised report form by DTs. At the same time, feedback from teachers and DTs by means of FGD and parents by means of in-depth interviews will be collected to assess the acceptability, feasibility (facilitators and barriers), effectiveness, and suggestions for improvement in implementing the SIMSP. The researchers will visit the preschools 3 times over the 6-month period to monitor the implementation fidelity at each preschool. Variations in the implementation process between preschools are minimised through discussion and support.

##### Plans for communicating important protocol amendments to relevant parties (e.g. trial participants, ethical committees) {25}

Any modifications to the protocol which may affect the conduct of the study, potential benefits to the children including changes in the study objectives, study procedures, or significant administrative aspects will require a formal amendment to the protocol. Such amendment must be agreed by the research team, the DTs, teachers, and parents involved in the trial. Resubmission of the protocol to the ethics committee for re-approval of ethics prior to implementation will be carried out. The senior dental officer of the Kampar district will be notified.

##### Dissemination plans {31a}

Each publication or abstract of the trial submitted to a research journal or a conference must be assessed and agreed by the research team. The study results will be released to the participating preschools, parents, Kampar main dental clinic, Perak State Health Department, and the Oral Health Programme, Ministry of Health.

## Discussion

The National Oral Health Survey of Preschool Children in 2005 and 2015 showed that caries prevalence among 5-year-old children in Malaysia were high at 76.2% and 71.3%, respectively [6, 7]. At the same time, the percentage of 5-year-old children with visible plaque in the 2015 survey was high at 60% [7]. These findings indicate that more should be done to improve the existing POHP to control caries and dental plaque in this age group.

Acknowledging the fact that young children’s life is dependent on significant others, the delivery of the existing POHP by DTs focusing on children alone will not be effective because young children’s oral health behaviours are very much influenced by their parents/caregivers as well as teachers [11, 12, 21]. Parental factors are important in determining the oral hygiene level and caries risk of children. Therefore, what parents do to influence their child’s oral health behaviours will determine their child’s future oral health [22]. At the same time, innovative school-based oral health education programmes especially designed for preschool children which incorporates parental and teachers’ involvement are essential to improve oral health behaviours and oral health status of preschool children [23, 24]. In this study, we have developed the SIMSP to incorporate DTs, teachers, and parents in children’s oral health with the view to improve the current POHP and children’s oral health in the long term.

A cluster RCT allows for statistical analysis of the potential effectiveness of the SIMSP intervention to control dental plaque and improve oral health behaviours of children [25–27]. This trial provides practical and methodological means for implementing community-based studies, especially when the intervention requires changes in behaviours and intends an effect at preschool level.

The protocol has its strengths and weaknesses. The multi-sectoral and multi-disciplinary collaborations from academia, oral healthcare services, parents, and teachers provide the researchers with a wide variety of interests, experience and perspectives on the SIMSP. This collaboration provides a better engagement between different stakeholders involved in the study. Also, randomisation at preschool level will reduce contamination of intervention packages. As the intervention is not yet formally incorporated into the preschool curriculum, the execution of the oral health lessons in class and daily toothbrushing at school will depend highly on the teacher’s efforts and commitment in conducting the activities. The challenge also lies in parents’ willingness to monitor child’s tooth brushing at home.

The process evaluation of the SIMSP provides an opportunity to assess the implementation aspects of the SIMSP from the stakeholders’ point of view. The programme may be improved further by addressing the barriers and suggestions put forward by the stakeholders. This study protocol caters to the recommendation for better delivery of preschool oral healthcare programme and to conduct scientific evaluation of all future interventions for improving oral health care for young children.

### Trial status

Recruitment of preschools was completed in February 2019 and recruitment of children was completed by April 2019. The primary outcome variable at 6-month follow-up was collected in October 2019. The quantitative data of the secondary outcome variables were collected in November 2019. Qualitative data collection for the secondary outcome variables is still ongoing; the FGD and in-depth interviews were stopped in February 2020 due to the movement control order enforced by the government in response to the Covid-19 pandemic, and are expected to resume in August 2020. Online FGD and in-depth interviews are not feasible as many parents and teachers in Kampar district do not have access to internet connection at home. Quantitative data entry and cleaning have been completed but multi-level data analysis is still ongoing due to the Covid-19 pandemic. The results of the trial are expected to be released to the stakeholders when all the analysis of the primary and secondary outcome variables have been completed.

## Data Availability

All investigators will be given access to the data sets. Data will be kept at the Faculty of Dentistry, University of Malaya, 50603, Kuala Lumpur, Malaysia.

## Abbreviations

MOH: Ministry of Health
POHP: Preschool oral healthcare programme
OHE: Oral health education
DTs: Dental therapists
SIMSP: Senyuman Indah Milik Semua Programme
OHL: Oral health literacy
ppm: Parts per million
CRA: Caries risk assessment
FGD: Focus group discussion
Malay-DHLAI: Malay version of Dental Health Literacy Assessment Instrument
OHK: Oral Health Knowledge
SM: Skills and Motivation
